# Systematic Theoretical Study on Structural, Stability, Electronic, and Spectral Properties of Si_2_${\text{Mg}}_{\text{n}}^{\text{Q}}$ (*Q* = 0, ±1; *n* = 1–11) Clusters of Silicon-Magnesium Sensor Material

**DOI:** 10.3389/fchem.2019.00771

**Published:** 2019-11-12

**Authors:** Ben-Chao Zhu, Ping-Ji Deng, Lu Zeng

**Affiliations:** ^1^School of Public Health and Management, Hubei University of Medicine, Shiyan, China; ^2^Center for Environment and Health in Water Source Area of South-to-North Water Diversion, Hubei University of Medicine, Shiyan, China; ^3^College of Materials Science and Engineering, Chongqing University, Chongqing, China

**Keywords:** silicon-magnesium sensor material, Si_2_Mg_n_^0,±1^ clusters, geometrical structures, electronic properties, spectral properties

## Abstract

By using CALYPSO searching method and Density Functional Theory (DFT) method at the B3LYP/6-311G (d) level of cluster method, a systematic study of the structures, stabilities, electronic and spectral properties of Si_2_${\text{Mg}}_{\text{n}}^{\text{Q}}$ (*n* = 1–11; *Q* = 0, ±1) clusters of silicon-magnesium sensor material, is performed. According to the calculations, it was found that when *n* > 4, most stable isomers in Si_2_${\text{Mg}}_{\text{n}}^{\text{Q}}$ (*n* = 1–11; *Q* = 0, ±1) clusters of silicon-magnesium sensor material are three-dimensional structures. Interestingly, although large size Si_2_${\text{Mg}}_{\text{n}}^{\text{Q}}$ clusters show cage-like structures, silicon atoms are not in the center of the cage, but tend to the edge. The Si_2_${\text{Mg}}_{1,5,6,8}^{-1}$ and Si_2_${\text{Mg}}_{13,4,7,9,10}^{+1}$ clusters obviously differ to their corresponding neutral structures, which are in good agreement with the calculated values of VIP, AIP, VEA, and AEA. |VIP-VEA| values reveal that the hardness of Si_2_Mg_n_ clusters decreases with the increase of magnesium atoms. The relative stabilities of neutral and charged Si_2_${\text{Mg}}_{\text{n}}^{\text{Q}}$ (*n* = 1–11; *Q* = 0, ±1) clusters of silicon-magnesium sensor material is analyzed by calculating the average binding energy, fragmentation energy, second-order energy difference and HOMO-LUMO gaps. The results reveal that the Si_2_${\text{Mg}}_{3}^{0}$, Si_2_${\text{Mg}}_{3}^{-1}$, and Si_2_${\text{Mg}}_{3}^{+1}$clusters have stronger stabilities than others. NCP and NEC analysis results show that the charges in Si_2_${\text{Mg}}_{\text{n}}^{\text{Q}}$ (*n* = 1–11; *Q* = 0, ±1) clusters of silicon-magnesium sensor material transfer from Mg atoms to Si atoms except for Si_2_${\text{Mg}}_{1}^{+1}$, and strong sp hybridizations are presented in Si atoms of Si_2_${\text{Mg}}_{\text{n}}^{\text{Q}}$ clusters. Finally, the infrared (IR) and Raman spectra of all ground state of Si_2_${\text{Mg}}_{\text{n}}^{\text{Q}}$ (*n* = 1–11; *Q* = 0, ±1) clusters of silicon magnesium sensor material are also discussed.

## Introduction

Silicon and magnesium are abundant elements on the earth and are widely used in sensor industry. In particular, silicon, as the main material of semiconductor sensors, has always been the research frontier in the field of sensors. As the only stable compound in Mg-Si binary system, Mg_2_Si, which has the characteristics of high melting point, high hardness, high modulus of elasticity and environmentally friendly, is an *n*-type semiconductor material with a band gap of 0.68–1.03 eV (Atanassov and Baleva, 2007). There are many experimental and theoretical studies on silicon-magnesium sensor materials. For example, theoretically, Morris et al. (1958) first used graphite crucible to melt stoichiometric components to prepare high purity single crystal Mg_2_Si materials, they found the band gap of Mg_2_Si is 0.78 eV. Aymerich and Mula (2010) and Imai et al. (2003) studied the band structure of Mg_2_Si using empirical and first-principles pseudopotentials, respectively. Chen et al. (2010) studied the band structure of Mg_2_Si and doped Ag, Al elements by using the first-principles pseudopotential plane wave method based on density functional theory (DFT). By using DFT, they obtained the real part, imaginary part and Photoconductivity of Mg_2_Si dielectric function as a function of photon energy. Experimentally, the main work on Mg_2_Si is focused on the preparation of thin film materials. Wittmer et al. (1979) was the first to fabricate Mg_2_Si semiconductor thin films on Si (111) substrates by evaporating Mg atoms films with different thicknesses using an electron gun at a speed of about 40Å/s in vacuum. Boher et al. (1992) used radio frequency magnetron sputtering technology to sputter Mg_2_Si targets onto glass materials and Si (111) substrates, and obtained amorphous Mg_2_Si films. Song et al. (2003) used pulsed laser deposition (PLD) method to grow Mg_2_Si crystal semiconductor thin films nearly 380 nanometers thick on stainless steel substrates at 500° annealing temperature.

All the above theoretical and experimental studies have greatly enriched the research results on the properties of silicon-magnesium sensor material. However, these studies have not touched the fundamental problem, how do the physical and chemical properties of silicon-magnesium compounds change from small systems (several or dozens of atoms) to large systems? Fortunately, small clusters provide a new way to study this system, which can provide insight into the strength and properties of metal bonds (Ju et al., 2015; Sun et al., 2017, 2018; Bole et al., 2018). Cluster material scale is a concept of nanomaterials. It is a relatively stable micro or sub-micro aggregate composed of several or even thousands of atoms, molecules or ions. Its physical and chemical properties usually vary with the number of atoms contained. Cluster studies have successfully helped us to in-depth understand the structure, stability, electronic states and spectral properties of many materials (Jin et al., 2015a,b; Xia et al., 2015; Xing et al., 2016a,b). There are many reports about sensor material study by using cluster method. For example, Yang et al. (2006) used full-muffin-tin-orbital molecular-dynamics (FP-LMTO-MD) method to study the electronic and geometric structures of Ga_n_As_n_ (*n* = 4, 5, 6) cluster ions. They found that some of the lowest energy structures for the cluster ions are different from those of the corresponding neutral clusters. Dmytruk et al. (2009) produced zinc oxide clusters by laser ablation of bulk powder zinc peroxide in vacuum and studied them by time-of-flight mass spectrometry. By comparing the experimental results with the theoretical calculations of clusters, the most stable structure of (ZnO)n clusters was verified at *n* = 34, 60, and 78.

However, most of the studies on sensor material clusters are carried out in a crystal growth mode, such as AsGa and ZnO, where the number of different atoms increases in harmony. In this paper, doped clusters will be used to study the materials of silicon-magnesium sensors. To be exact, we doped a small amount of silicon into magnesium element, which increased the number of magnesium atoms around two silicon atoms from 1 to 11, and made them neutral charged, negative charged and positive charged, respectively. Then, we will study the structure, stability, electronic and spectral properties of Si_2_${\text{Mg}}_{\text{n}}^{\text{Q}}$ (*n* = 1–11; *Q* = 0, ±1) clusters of silicide-magnesium materials in detail. The paper is organized as follows: Section Computation Methods describes the computational details, the results are presented and completely discussed in section Results and discussions, and the final conclusions are summarized in section Conclusion.

## Computation Methods

All structural optimization and infrared Raman spectrum analysis are carried out by using DFT at B3LYP/6-311G (d) basis set level in Gauss 09 program package (Frisch et al., 2014). In order to find the lowest energy state structure of Si_2_${\text{Mg}}_{\text{n}}^{\text{Q}}$ (*n* = 1–11; *Q* = 0, ±1) clusters of silicon-magnesium sensor material, it is necessary to prepare enough initial configurations of Si_2_Mg_n_ clusters. We used the particle swarm optimization (CALYPSO) method (Wang et al., 2010, 2012; Lv et al., 2012) to get the initial structures of pure magnesium clusters. Then, replacing any two Mg atoms with Si atom in the initial Mg_n_ clusters' structures. CALYPSO method has successfully predicted structures for various systems ranging from clusters to crystal structures (Lu et al., 2013, 2017, 2018; Lu and Chen, 2018; Xiao et al., 2019). In the process of geometric optimization in Gauss 09 package, for neutral clusters, the spin multiplicity of electrons takes into account 1, 3, 5 states, while for charged clusters, it is 2, 4, 6 states, and there is no constraint on the symmetry. Finally, if the optimization results include virtual frequencies, the coordinates of the virtual mode are relaxed until the real local minimum is obtained. On the basis of eliminating imaginary frequency, the potential energy of all optimized ground state structures will reach absolute local minimum.

In order to prove the reliability of the selected B3LYP/6-311G (d) basis set level, the calculated bond length, vibrational frequency, vertical ionization potential (VIP) and vertical electron affinity (VEA)of the neutral Mg_2_, Si_2_, SiMg clusters by using different methods at the same 6-311G (d) basis set are shown in Table 1. As showed in Table 1, the calculated values *r*(Mg_2_) = 3.93 Å, ω(Mg_2_) = 44.96 cm^−1^, *r*(Si_2_) = 2.17 Å, ω(Si_2_) = 540.82 cm^−1^, VIP(Si_2_) = 9.13 eV, and VEA(Si_2_) = 2.02 eV, these conclusions are quite agree with the existed experimental results (Huber, 1979; de Heer et al., 1987; Kitsopoulos et al., 1991; Ruette et al., 2005).

**Table 1:** Calculated values of bond length *r* (Å), frequency ω (cm^−1^), vertical ionization potential VIP (eV) and vertical electron affinity VEA (eV) for the Mg_2_, Si_2_, and SiMg clusters by different methods.

**Methods**	**Mg**_****2****_				**Si**_****2****_				**SiMg**			
	***r***	**ω**	**VIP**	**VEA**	***r***	**ω**	**VIP**	**VEA**	***r***	**ω**	**VIP**	**VEA**
B3LYP	3.93	44.96	8.16	0.43	2.17	540.82	9.13	2.02	2.57	288.31	6.77	0.61
B3PW91	3.61	85.29	6.20	0.22	2.31	476.71	8.53	2.79	2.54	325.98	5.84	1.70
PBE	2.78	263.51	4.75	1.68	2.18	531.49	8.15	2.08	2.56	311.01	6.91	0.96
BPV86	2.78	259.56	7.71	0.69	2.18	527.65	7.84	2.08	2.55	306.74	7.94	1.32
MP1PW91	3.60	88.05	6.16	0.21	2.30	484.06	8.54	2.79	2.54	327.96	5.80	1.71
Expt	3.89^a^	45^a^	–	–	2.25^b^	511^b^	>8.49^c^	2.176 ± 0.002^d^	–	–	–	–

a *Ruette et al. (2005)*.

b *Huber (1979)*.

c *de Heer et al. (1987)*.

d *Kitsopoulos et al. (1991)*.

## Results and Discussions

### Geometrical Structures of Si_2_${\text{Mg}}_{\text{n}}^{\text{Q}}$(*n* = 1–11; *Q* = 0, ±1) Clusters of Silicon-Magnesium Sensor Material

**Figure 1:**
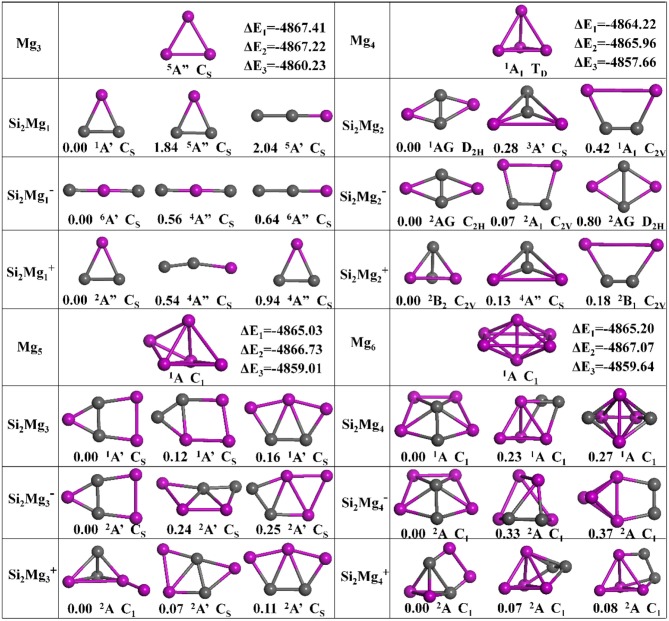
Optimized geometries of Mg_n+2_ and Si_2_${\text{Mg}}_{\text{n}}^{\text{Q}}$ (*n* = 1–4; *Q* = 0, ±1) clusters of silicon-magnesium sensor material at B3LYP/6-311+G(d) level. The pink and gray balls present the Mg and Si atoms, respectively.

**Figure 2:**
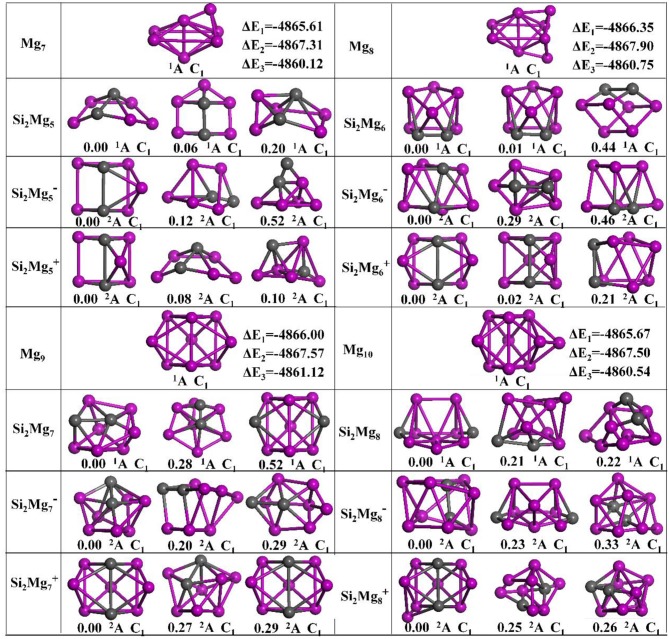
Optimized geometries of Mg_n+2_ and Si_2_${\text{Mg}}_{\text{n}}^{\text{Q}}$ (*n* = 5–8; *Q* = 0, ±1) clusters of silicon-magnesium sensor material at B3LYP/6-311+G(d) level. The pink and gray balls present the Mg and Si atoms, respectively.

**Figure 3:**
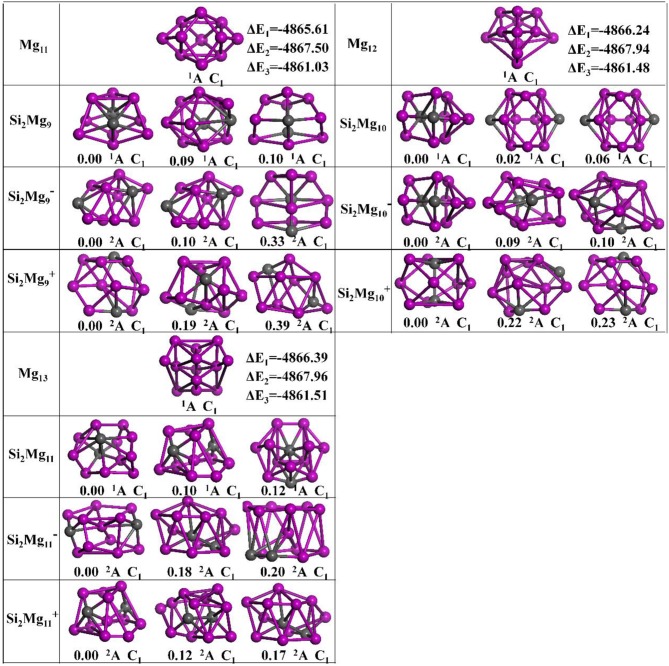
Optimized geometries of Mg_n+2_ and Si_2_${\text{Mg}}_{\text{n}}^{\text{Q}}$ (*n* = 9–11; *Q* = 0, ±1) clusters of silicon-magnesium sensor material at B3LYP/6-311+G(d) level. The pink and gray balls present the Mg and Si atoms, respectively.

The geometries of Si_2_${\text{Mg}}_{\text{n}}^{\text{Q}}$ (*n* = 1–11; *Q* = 0, ±1) clusters of silicon-magnesium sensor material are optimized by using the computational method in section Computation Methods. Due to the existence of so many initial structures, the relative energies of all the initial isomers with different spin multiplicities are optimized, but only the lowest energies and a few low-lying energy isomers are given in Figures 1–3. In addition, in Figures 1–3, in order to compare the effect of Si-doped Mg clusters on the original structure of pure Mg clusters, we also list the lowest energy state structure Mg_n+2_ (*n* = 1–11) of pure Mg clusters optimized by the same method, while the lowest energy state and two metastable structures of neutral Si_2_${\text{Mg}}_{\text{n}}^{0}$, anionic Si_2_${\text{Mg}}_{\text{n}}^{-1}$, cationic Si_2_${\text{Mg}}_{\text{n}}^{+1}$ (*n* = 1–11) clusters are given. Under each isomer structure, there are three information about the energy difference between the metastable structure and the lowest energy state structure, the symmetry, and the electronic spin state. So, the first structure of Si_2_${\text{Mg}}_{\text{n}}^{\text{Q}}$ clusters are all labeled as 0.00 eV, indicating that this structure is the lowest energy state. The latter two are two metastable structures, and the energy difference with the lowest energy state is directly expressed as a non-zero value. It is noteworthy that when *n* is determined, there are three energy differences on the right side of the lowest energy structure of Mg_n+2_, they are ΔE_1_ = E(Si_2_${\text{Mg}}_{\text{n}}^{0}$)–E(Mg_n+2_), ΔE_2_ = E(Si_2_${\text{Mg}}_{\text{n}}^{-1}$)–E(Mg_n+2_), and ΔE_3_ = E(Si_2_${\text{Mg}}_{\text{n}}^{+1}$)–E(Mg_n+2_), notably, E means the ground state energy. Since there are too many structures, we first give a brief introduction to each structure, and then analyze and discuss their growth patterns shortly below.

*n* = *1*: *Si*_2_*Mg*_1_*, Si*_2_${Mg}_{1}^{-1}$*, Si*_2_${Mg}_{1}^{+1}$*, and Mg*_3_

The lowest energy structure of neutral Si_2_Mg1 with spin singlet and C_S_ symmetry is an isosceles triangle, which are the similar as the ground state of cationic Si_2_${\text{Mg}}_{1}^{+1}$ and pure Mg_3_ clusters. For anionic Si_2_${\text{Mg}}_{1}^{-1}$, the linear chain (C_S_, ^6^A') in which the Mg atom is in the middle position is found to be the most stable isomer. For metastable isomers, two triangular structures for Si_2_${\text{Mg}}_{1}^{+1}$, two linear chain structures for Si_2_${\text{Mg}}_{1}^{-1}$, and one triangular, one linear chain structures for neutral Si_2_Mg1.

*n* = *2*: *Si*_2_*Mg*_2_*, Si*_2_${Mg}_{2}^{-1}$*, Si*_2_${Mg}_{2}^{+1}$*, and Mg*_4_

The ground states of Si_2_Mg2 (D_2H_, ^1^AG) and Si_2_${\text{Mg}}_{2}^{-1}$ (C_2H_, ^2^AG) are parallelograms with a little different shapes. Replacing any two Mg atoms with Si atoms in the tetrahedral structure of Mg_4_ (T_D_, ^1^A_1_) forms the lowest energy isomer structure of Si_2_${\text{Mg}}_{2}^{+1}$ (C_2V_, ^2^B_2_). All metastable isomers are planar structures, such as trapezoids, triangles and parallelograms.

*n* = *3*: *Si*_2_*Mg*_3_*, Si*_2_${Mg}_{3}^{-1}$*, Si*_2_${Mg}_{3}^{+1}$*, and Mg*_5_

It is impossible to replace two magnesium atoms in the ground state structure of Mg_5_ (C_1_, ^1^A) with silicon atoms to directly form any Si_2_MgQ 3 (*Q* = 0, ±1) cluster structure. But the lowest energy isomer structures of Si_2_Mg3 (C_S_, ^1^A') and Si_2_${\text{Mg}}_{3}^{-1}$ (C_S_, ^2^A') can be formed by the second metastable isomer structure of Si_2_Mg2, in where attracting a Mg atom in the same plane outside the trapezoidal silicon-silicon bond. The lowest energy isomer structure of Si_2_${\text{Mg}}_{3}^{+1}$ (C_1_, ^2^A) is formed by the ground state of Si_2_${\text{Mg}}_{2}^{+1}$ with a magnesium cap at the top of a magnesium atom. In addition, all metastable isomers exhibit planar structures.

*n* = *4*: *Si*_2_*Mg*_4_*, Si*_2_${Mg}_{4}^{-1}$*, Si*_2_${Mg}_{4}^{+1}$*, and Mg*_6_

The lowest energy structure of Mg_6_ (C_1_, ^1^A) is an octahedron. When the two magnesium atoms at the octahedron vertex are replaced by silicon atoms and the lower silicon atoms float up to the plane where the four magnesium atoms are located, the lowest energy state structures of Si_2_Mg_4_ (C_1_, ^1^A) and Si_2_${\text{Mg}}_{4}^{-1}$ (C_1_, ^2^A) are formed. The ground state structure of Si_2_${\text{Mg}}_{4}^{+1}$ (C_1_, ^2^A) can be formed by the ground state of Si_2_${\text{Mg}}_{2}^{+1}$attrcating a Mg-Mg bond parallel to the Si-Si bond. All metastable isomers are three-dimensional structures and are directly related to the structure of isomers with small *n* values.

*n* = *5*: *Si*_2_*Mg*_5_*, Si*_2_${Mg}_{5}^{-1}$*, Si*_2_${Mg}_{5}^{+1}$*, and Mg*_7_

The ground state structure of Mg_7_ (C_1_, ^1^A) can be directly formed from Mg_6_ with a magnesium atom cap on one side of the octahedron. The ground state structures of Si_2_${\text{Mg}}_{5}^{-1}$ (C_1_, ^2^A) and Si_2_${\text{Mg}}_{5}^{+1}$ (C_1_, ^2^A) are similar, their main body is a triangular prism with a magnesium-silicon-magnesium triangle at the top and bottom, and then a magnesium atom cap at different distances from the side. The lowest energy structure of neutral Si_2_Mg_5_ (C_1_, ^2^A) is formed when the ground sate structure of Si_2_Mg_4_ attracting one magnesium atom. It is easy to see that the first metastable structure of cationic Si_2_${\text{Mg}}_{5}^{+1}$ is the lowest energy state structure of neutral Si_2_Mg_5_. Interestingly, the difference between the first metastable state structure of neutral Si_2_Mg_5_ and the lowest energy state structure of anionic Si_2_${\text{Mg}}_{5}^{-1}$ is the orientation of the cap with magnesium atom, the former at the bottom and the latter at the side.

*n* = *6*: *Si*_2_*Mg*_6_*, Si*_2_${Mg}_{6}^{-1}$*, Si*_2_${Mg}_{6}^{+1}$*, and Mg*_8_

The lowest energy structure of Mg_8_ (C_1_, ^1^A) is formed by adding a Mg atom cap to the up down mirror symmetry of Mg_7_. When adding a Mg atom cap to the right left mirror symmetry of the lowest energy structure of Si_2_${\text{Mg}}_{5}^{+1}$, the ground state structures of Si_2_${\text{Mg}}_{6}^{+1}$ (C_1_, ^2^A) is formed. The lowest energy structure of neutral Si_2_Mg_6_ (C_1_, ^1^A) is as the same as its first metastable structure. The ground state of Si_2_${\text{Mg}}_{6}^{-1}$(C_1_, ^2^A) is an irregular polyhedral cylinder, but its metastable state structures show certain irregularity.

*n* = *7*: *Si*_2_*Mg*_7_*, Si*_2_${Mg}_{7}^{-1}$*, Si*_2_${Mg}_{7}^{+1}$*, and Mg*_9_

The lowest energy state structure of the cationic Si_2_${\text{Mg}}_{7}^{+1}$ (C_1_, ^2^A), which is as the same as its second metastable state structure, can be formed by substituting the upper and lower mirror symmetrical Mg atoms for the silicon atoms in the lowest energy state Mg_9_ (C_1_, ^1^A) structure. The ground state of Si_2_${\text{Mg}}_{7}^{-1}$ (C_1_, ^2^A) is similar as the first metastable state structure Si_2_${\text{Mg}}_{7}^{+1}$. The lowest energy structure of the neutral Si_2_Mg_7_ (C_1_, ^2^A) has the same main body as the ground state structure of Si_2_Mg_4_. Interestingly, the second metastable state structure of Si_2_Mg_7_ is similar as the ground state structure of Si_2_${\text{Mg}}_{7}^{+1}$, the only difference is the two silicon atoms are bonded from top to bottom to left.

*n* = *8*: *Si*_2_*Mg*_8_*, Si*_2_${Mg}_{8}^{-1}$*, Si*_2_${Mg}_{8}^{+1}$*, and Mg*_10_

The lowest energy state structure of the Mg_10_ (C_1_, ^1^A) is formed by Mg_9_ with a magnesium atom on right side. The ground state structure of Si_2_${\text{Mg}}_{8}^{+1}$ (C_1_, ^2^A) can be formed by ground state structure of Si_2_${\text{Mg}}_{7}^{+1}$ with a magnesium cap on left-down side. The lowest energy state structure of neutral Si_2_Mg_8_ (C_1_, ^2^A) is similar as the first metastable state structure of Si_2_${\text{Mg}}_{8}^{-1}$. The ground state of Si_2_${\text{Mg}}_{8}^{-1}$(C_1_, ^2^A) is cage-like structure with one silicon atom trapped on the upper surface. Interestingly, other metastable state structures also present cage-like structures.

*n* = *9*: *Si*_2_*Mg*_9_*, Si*_2_${Mg}_{9}^{-1}$*, Si*_2_${Mg}_{9}^{+1}$*, and Mg*_11_

When Mg_10_ attracting a magnesium on the left side, it is the lowest energy structure of Mg_11_ (C_1_, ^1^A). From *n* = 9, it is easy found that no structure of Si_2_${\text{Mg}}_{\text{n}}^{\text{Q}}$ (*Q* = 0, ±1) can be formed by substituting two magnesium atoms for silicon atoms in Mg_n+2_. The ground state of Si_2_${\text{Mg}}_{9}^{-1}$ (C_1_, ^2^A) is similar as its first metastable state structure. They can be formed based on the first metastable structure of Si_2_${\text{Mg}}_{8}^{+1}$ with a Mg atomic cap. The ground state structure of neutral Si_2_Mg_9_ (C_1_, ^2^A) is similar as its second metastable structure and the second metastable structure of Si_2_${\text{Mg}}_{9}^{-1}$. The lowest energy state structure of Si_2_${\text{Mg}}_{9}^{+1}$ (C_1_, ^2^A) is a complex 3D cage-like structure based on the second metastable state of Si_2_Mg_4_ with attracting more five Mg atoms.

*n* = *10*: *Si*_2_*Mg*_10_*, Si*_2_${Mg}_{1}^{-1}$*0, Si*_2_${Mg}_{1}^{+1}$*0, and Mg*_12_

The ground state structures of neutral Si_2_Mg_10_ (C_1_, ^1^A) and Si_2_${\text{Mg}}_{1}^{-1}$0 (C_1_, ^2^A) are the same and can be formed by the lowest energy state structure of Si_2_${\text{Mg}}_{9}^{+1}$ with a magnesium cap. The lowest energy state structure of Si_2_${\text{Mg}}_{1}^{+1}$0 (C_1_, ^2^A) is formed by the ground state structure of Si_2_${\text{Mg}}_{8}^{-1}$ with adding two magnesium atoms. All the metastable structures present 3D structures, and some of them can easily be found to be associated with the cluster structure discussed earlier. For example, the metastable structure of neutral Si_2_Mg_10_ can be formed by the ground state of neutral Si_2_Mg_8_ with adding two Mg atoms.

*n* = *11*: *Si*_2_*Mg*_11_*, Si*_2_${Mg}_{1}^{-1}$*1, Si*_2_${Mg}_{1}^{+1}$*1, and Mg*_13_

The lowest energy structures of Si_2_Mg_11_ (C_1_, ^1^A), Si_2_${\text{Mg}}_{1}^{-1}$1(C_1_, ^2^A), Si_2_${\text{Mg}}_{1}^{+1}$1(C_1_, ^2^A) show cage structures, but no silicon atom located the cage center. By using the ground state structure of Si_2_${\text{Mg}}_{9}^{-1}$ with adding two magnesium atoms, the lowest energy structure of Si_2_${\text{Mg}}_{1}^{-1}$1(C_1_, ^2^A) is got. The ground state structure of Si_2_Mg_11_ (C_1_, ^1^A) can be formed by the first metastable structure of Si_2_Mg_9_ with two more magnesium attracted. The lowest energy structure of Si_2_${\text{Mg}}_{1}^{+1}$1(C_1_, ^2^A) is the same as the first metastable structure of Si_2_Mg_11_, and they are quite similar as the first metastable structure of Si_2_${\text{Mg}}_{9}^{+1}$. Other metastable structures exhibit 3D cage-like structure.

#### Energy Difference Between Structures

As shown in Figures 1–3, the energy differences ΔE_1_ (from −4867.41 to −4864.22 eV), ΔE_2_ (from −4867.96 to −4865.96 eV), and ΔE_3_ (from −4861.51 to −4857.66 eV) are quite stable and reasonable. Because the energy difference between the free neutral Si_2_ and Mg_2_, E(Si_2_)–E(Mg_2_) = −4864.42 eV, is quite near to the ΔE_1._ In addition, ΔE_2_ < ΔE_1_ < ΔE_3_ is consistent with the following conclusion: if the neutral charged cluster is negatively charged, the cluster will lose energy, and if the neutral charged cluster is positively charged, the cluster will get energy. In addition, the energy differences between all metastable state structures and their corresponding ground state structures are also listed under each metastable state structure, they are all very small (from 0.01 to 2.04 eV) and reasonable.

#### Growth Pattern

According to the structural characteristics of the lowest energy state structures mentioned above, the growth mechanism of Si_2_${\text{Mg}}_{\text{n}}^{\text{Q}}$ (*n* = 1–11; *Q* = 0, ±1) clusters of silicon-magnesium sensor material can be summarized as following: (i) The lowest energy state Si_2_${\text{Mg}}_{\text{n}}^{\text{Q}}$ clusters favor 3D and low spin multiplicity for *n* = 4–11. (ii) Compared with neutral Si_2_Mg_n_ clusters, charged Si_2_${\text{Mg}}_{\text{n}}^{\pm 1}$ clusters formed when they get or lose electrons will change their structures in most cases. (iii) Larger size clusters Si_2_${\text{Mg}}_{\text{n}}^{0\pm 1}$ show cage-like geometries, but silicon atoms are not in the center of the cage, but tend to the edge, which is different from some reports (Zhang et al., 2015). This may be related to the distribution of electrons outside the nucleus of magnesium and silicon atoms. Through the above structure optimization, we can find that the shortest chemical bond length of clusters tends to be smaller when silicon doped with magnesium. Table 2 shows the shortest chemical bond lengths of Mg-Mg, Si-Si, Si-Mg for all Si_2_Mg_n_ clusters as the number of magnesium atoms increases. For comparison, Table 2 also lists the shortest chemical bond lengths of Mg-Mg clusters with corresponding atomic numbers of pure magnesium clusters. From Table 2, it can be seen clearly that silicon doping into magnesium can indeed make the cluster structure more compact when the total number of atoms is the same.

**Table 2:** The shortest bond length (Å) of Mg_n+2_, neutral and charged Si_2_${\text{Mg}}_{\text{n}}^{\text{Q}}$ (*n* = 1–11; *Q* = 0, ±1) clusters.

**Clusters**	**The shortest bond length (Å)**			**Clusters**	**The shortest bond length (Å)**
	**Anionic**	**Cationic**	**Neutral**		
Si_2_Mg_1_	d_Si−Si_ = 5.27	d_Si−Si_ = 2.31	d_Si−Si_ = 2.21	Mg_3_	d_Mg−Mg_ = 2.91
	d_Si−Mg_ = 2.63	d_Si−Mg_ = 2.70	d_Si−Mg_ = 2.54		
Si_2_Mg_2_	d_Si−Si_ = 2.37	d_Si−Si_ = 2.47	d_Si−Si_ = 2.22	Mg_4_	d_Mg−Mg_ = 3.17
	d_Si−Mg_ = 2.66	d_Si−Mg_ = 2.66	d_Si−Mg_ = 2.79		
	d_Mg−Mg_ = 4.76	d_Mg−Mg_ = 2.99	d_Mg−Mg_ = 5.13		
Si_2_Mg_3_	d_Si−Si_ = 2.25	d_Si−Si_ = 2.21	d_Si−Si_ = 2.32	Mg_5_	d_Mg−Mg_ = 3.45
	d_Si−Mg_ = 2.67	d_Si−Mg_ = 2.66	d_Si−Mg_ = 2.53		
	d_Mg−Mg_ = 3.01	d_Mg−Mg_ = 2.96	d_Mg−Mg_ = 3.00		
Si_2_Mg_4_	d_Si−Si_ = 2.23	d_Si−Si_ = 2.75	d_Si−Si_ = 2.27	Mg_6_	d_Mg−Mg_ = 3.00
	d_Si−Mg_ = 2.77	d_Si−Mg_ = 2.59	d_Si−Mg_ = 2.68		
	d_Mg−Mg_ = 3.03	d_Mg−Mg_ = 2.99	d_Mg−Mg_ = 2.93		
Si_2_Mg_5_	d_Si−Si_ = 2.32	d_Si−Si_ = 2.59	d_Si−Si_ = 2.22	Mg_7_	d_Mg−Mg_ = 3.15
	d_Si−Mg_ = 2.69	d_Si−Mg_ = 2.58	d_Si−Mg_ = 2.60		
	d_Mg−Mg_ = 3.05	d_Mg−Mg_ = 2.98	d_Mg−Mg_ = 2.88		
Si_2_Mg_6_	d_Si−Si_ = 2.33	d_Si−Si_ = 2.56	d_Si−Si_ = 2.74	Mg_8_	d_Mg−Mg_ = 3.14
	d_Si−Mg_ = 2.65	d_Si−Mg_ = 2.64	d_Si−Mg_ = 2.60		
	d_Mg−Mg_ = 2.99	d_Mg−Mg_ = 2.99	d_Mg−Mg_ = 2.87		
Si_2_Mg_7_	d_Si−Si_ = 2.29	d_Si−Si_ = 2.36	d_Si−Si_ = 2.23	Mg_9_	d_Mg−Mg_ = 3.12
	d_Si−Mg_ = 2.67	d_Si−Mg_ = 2.67	d_Si−Mg_ = 2.66		
	d_Mg−Mg_ = 3.03	d_Mg−Mg_ = 3.02	d_Mg−Mg_ = 2.90		
Si_2_Mg_8_	d_Si−Si_ = 2.39	d_Si−Si_ = 2.69	d_Si−Si_ = 5.15	Mg_10_	d_Mg−Mg_ = 3.04
	d_Si−Mg_ = 2.67	d_Si−Mg_ = 2.65	d_Si−Mg_ = 2.54		
	d_Mg−Mg_ = 3.01	d_Mg−Mg_ = 2.99	d_Mg−Mg_ = 2.89		
Si_2_Mg_9_	d_Si−Si_ = 5.02	d_Si−Si_ = 2.39	d_Si−Si_ = 5.72	Mg_11_	d_Mg−Mg_ = 3.08
	d_Si−Mg_ = 2.64	d_Si−Mg_ = 2.65	d_Si−Mg_ = 2.58		
	d_Mg−Mg_ = 2.98	d_Mg−Mg_ = 3.03	d_Mg−Mg_ = 2.92		
Si_2_Mg_10_	d_Si−Si_ = 5.90	d_Si−Si_ = 2.45	d_Si−Si_ = 5.84	Mg_12_	d_Mg−Mg_ = 3.09
	d_Si−Mg_ = 2.64	d_Si−Mg_ = 2.67	d_Si−Mg_ = 2.55		
	d_Mg−Mg_ = 3.03	d_Mg−Mg_ = 3.02	d_Mg−Mg_ = 2.88		
Si_2_Mg_11_	d_Si−Si_ = 5.17	d_Si−Si_ = 5.22	d_Si−Si_ = 6.04	Mg_13_	d_Mg−Mg_ = 3.03
	d_Si−Mg_ = 2.64	d_Si−Mg_ = 2.67	d_Si−Mg_ = 2.68		
	d_Mg−Mg_ = 2.96	d_Mg−Mg_ = 2.96	d_Mg−Mg_ = 2.93		

### The Relative Stabilities of Si_2_${\text{Mg}}_{\text{n}}^{\text{Q}}$(*n* = 1–11; *Q* = 0, ±1) Clusters of Silicon-Magnesium Sensor Material

In order to study the relativity stabilities of neutral and charged Si_2_${\text{Mg}}_{\text{n}}^{\text{Q}}$ (*n* = 1–11; *Q* = 0, ±1) clusters of silicon-magnesium sensor material, the average binding energy *E*_b_, fragmentation energy *E*_f_, the second-order energy differences Δ_2_*E*, and the HOMO-LUMO energy gap E_gap_ are calculated, which can be read as below:

(1) $$E_{b}\left(Si_{2}Mg_{n}\right)=\left[nE_{k}(Mg)+2E_{k}(Si)-E_{k}(Si_{2}Mg_{n})\right]/\text{(}n+2)$$

(2) $$\begin{array}{l} E_{b}\left(Si_{2}Mg_{n}^{\pm 1}\right)=[\left(n-1)E_{k}(Mg)+E_{k}(Mg^{\pm }\right)\\ \;+2E_{k}(Si)-E_{k}\left(Si_{2}Mg_{n}^{\pm 1}\right)]/\text{(}n\text{+2)} \end{array}$$

(3) $$E_{f}\left(Si_{2}Mg_{n}^{0,\pm 1}\right)=E_{k}\left(Si_{2}Mg_{n-1}^{0,\pm 1}\right)+E_{k}\left(Mg\right)-E_{k}\left(Si_{2}Mg_{n}^{0,\pm 1}\right)$$

(4) $$\begin{array}{l} \Delta_{2}E\left(Si_{2}Mg_{n}^{0,\pm 1}\right)=E_{k}\left(Si_{2}Mg_{n-1}^{0,\pm 1}\right)+E_{k}\left(Si_{2}Mg_{n+1}^{0,\pm 1}\right)\\ \;-2E_{k}\left(Si_{2}Mg_{n}^{0,\pm 1}\right) \end{array}$$

(5) $$E_{gap}\left(Si_{2}Mg_{n}^{0,\pm 1}\right)=E_{LUMO}\left(Si_{2}Mg_{n}^{0,\pm 1}\right)-E_{HOMO}\left(Si_{2}Mg_{n}^{0,\pm 1}\right)$$

*E*_k_ in Equations (1–4) are the total energy of the corresponding atom and ground state clusters. *E*_HOMO_ and *E*_LUMO_ in Equation (5) are the energies of highest occupied molecular orbital (HOMO) and the lowest unoccupied molecular orbital (LUMO).

**Figure 4:**
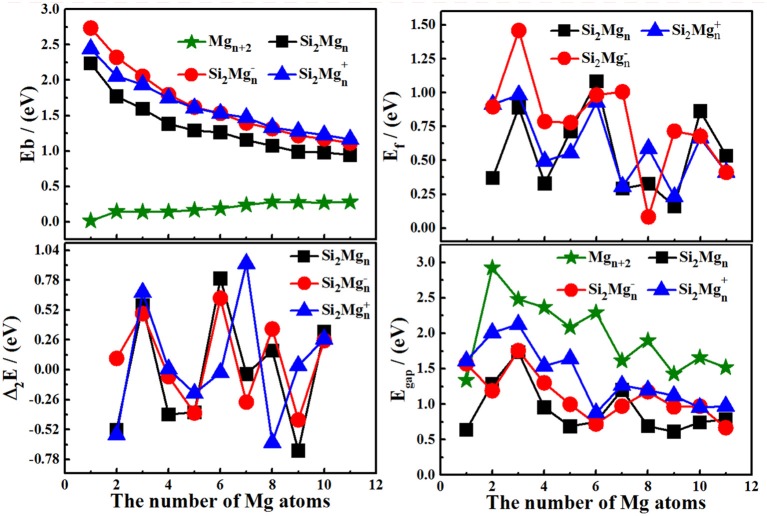
The size-dependent properties of E_*b*_, Δ_2_*E*, E_*f*_, and E_*gap*_ of the lowest-energy Si_2_${\text{Mg}}_{\text{n}}^{\text{Q}}$(*n* = 1–11; *Q* = 0, ±1) clusters of silicon-magnesium sensor material.

The motivation for comparing pure magnesium clusters must be explained here. Physically, the most ideal (simplest) silicon doping is to replace two magnesium atoms with silicon atoms in pure magnesium clusters, and then to optimize the structure. Therefore, comparing some properties of silicon-doped magnesium clusters, we always habitually compare pure magnesium clusters with the total number of corresponding atoms in our research. The size-dependent properties of *E*_b_, *E*_f_, Δ_2_*E*, and *E*_gap_ for the lowest energy state Si_2_${\text{Mg}}_{\text{n}}^{\text{Q}}$ (*n* = 1–11; *Q* = 0, ±1) clusters of silicon-magnesium sensor material are presented in Figure 4. We can summarize the properties as the following:

1. The *E*_b_ values of all Si_2_${\text{Mg}}_{\text{n}}^{\text{Q}}$ (*n* = 1–11; *Q* = 0, ±1) clusters of silicon-magnesium sensor material decrease followed by same tendency with the size increases, but the *E*_b_ values of pure Mg_n+2_ clusters are gradually increase. In addition, the *E*_b_ values of cationic Si_2_${\text{Mg}}_{\text{n}}^{+1}$ are always the highest, while the *E*_b_ values of neutral Si_2_${\text{Mg}}_{\text{n}}^{0}$ are the lowest all the time. It means that electron removal can enhance the chemical properties of Si_2_Mg_n_ clusters.
2. The *E*_f_ curves of neutral and charged Si_2_${\text{Mg}}_{\text{n}}^{\text{Q}}$ (*n* = 1–11; *Q* = 0, ±1) clusters of silicon-magnesium sensor material have a similar oscillating tendency. For neutral Si_2_${\text{Mg}}_{\text{n}}^{0}$ clusters, the stronger relative stability clusters are Si_2_${\text{Mg}}_{3}^{0}$, Si_2_${\text{Mg}}_{6}^{0}$, and Si_2_${\text{Mg}}_{10}^{0}$ based on the maxim of *E*_f_ values. For anionic Si_2_${\text{Mg}}_{\text{n}}^{-1}$ clusters, three significant maxima are found at *n* = 3, 7, 9, which indicate that Si_2_${\text{Mg}}_{3}^{-1}$, Si_2_${\text{Mg}}_{7}^{-1}$, and Si_2_${\text{Mg}}_{9}^{-1}$ clusters are the most stable clusters. For cationic Si_2_${\text{Mg}}_{\text{n}}^{+1}$ clusters, three local peaks can be found from the *E*_f_ curve, it means that Si_2_${\text{Mg}}_{3}^{+1}$, Si_2_${\text{Mg}}_{6}^{+1}$, Si_2_ ${\text{Mg}}_{8}^{+1}$ clusters are more stable than their neighbors.
3. The irregular oscillation behaviors are the most prominent feature of Δ_2_*E* curves of all Si_2_${\text{Mg}}_{\text{n}}^{\text{Q}}$ (*n* = 1–11; *Q* = 0, ±1) clusters of silicon-magnesium sensor material. The maxima are found at *n* = 3 for all Si_2_${\text{Mg}}_{\text{n}}^{\text{Q}}$ clusters, *n* = 6 and 8 for both neutral Si_2_${\text{Mg}}_{\text{n}}^{0}$ and anionic Si_2_${\text{Mg}}_{\text{n}}^{-1}$ clusters, *n* = 7 for cationic Si_2_${\text{Mg}}_{\text{n}}^{+1}$ clusters. It means that the Si_2_${\text{Mg}}_{3}^{-1}$, Si_2_${\text{Mg}}_{6}^{-1}$, Si_2_${\text{Mg}}_{8}^{-1}$, Si_2_${\text{Mg}}_{3}^{+1}$, Si_2_Mg+1 6, and Si_2_${\text{Mg}}_{7}^{+1}$ clusters have slightly stronger relative stabilities and have large abundances in mass spectroscopy in comparison with the corresponding neighbors. For neutral clusters, Si_2_${\text{Mg}}_{3}^{0}$, Si_2_${\text{Mg}}_{6}^{0}$, and Si_2_${\text{Mg}}_{8}^{0}$ clusters are more stable than other clusters.
4. The pure Mg_n+2_ clusters have the highest E_gap_ is an unexpected conclusion, because pure magnesium has higher chemical stability than silicon magnesium. For Si_2_${\text{Mg}}_{\text{n}}^{\text{Q}}$ (*n* = 1–11; *Q* = 0, ±1) clusters, the E_gap_ of cationic Si_2_${\text{Mg}}_{\text{n}}^{+1}$ clutters is always the higher one. It means that Si_2_${\text{Mg}}_{\text{n}}^{+1}$ clusters have higher chemical stability than the neutral and anionic Si_2_${\text{Mg}}_{\text{n}}^{\text{Q}}$ clusters. The curves of E_gap_ show that the maxima values appear at *n* = 3 for all Si_2_${\text{Mg}}_{\text{n}}^{\text{Q}}$ (*Q* = 0, ±1) clusters, *n* = 7 for both neutral Si_2_${\text{Mg}}_{\text{n}}^{0}$ and cationic Si_2_${\text{Mg}}_{\text{n}}^{+1}$, and *n* = 8 for anionic Si_2_${\text{Mg}}_{\text{n}}^{-1}$ clusters, which implies that the higher chemical stability clusters are Si_2_${\text{Mg}}_{3}^{0}$, Si_2_${\text{Mg}}_{3}^{-1}$, Si_2_${\text{Mg}}_{3}^{+1}$, Si_2_${\text{Mg}}_{7}^{0}$, Si_2_${\text{Mg}}_{7}^{+1}$, and Si_2_${\text{Mg}}_{8}^{-1}$.

Based on the discussions about *E*_b_, *E*_f_, Δ_2_*E*, and *E*_gap_, we can conclude that the magic numbers of neutral and charged Si_2_${\text{Mg}}_{\text{n}}^{\text{Q}}$ (*n* = 1–11; *Q* = 0, ±1) clusters of silicon-magnesium sensor material are Si_2_${\text{Mg}}_{3}^{0}$, Si_2_${\text{Mg}}_{3}^{-1}$, Si_2_${\text{Mg}}_{3}^{+1}$.

### The Charge Transfer of Si_2_${\text{Mg}}_{\text{n}}^{\text{Q}}$(*n* = 1–11; *Q* = 0, ±1) Clusters of Silicon-Magnesium Sensor Material

Natural charge population (NCP) and natural electron population (NEC) of clusters are two important parameters to study the localization of charges in clusters (Trivedi et al., 2014). In order to study internal charge transfer of neutral and charged Si_2_${\text{Mg}}_{\text{n}}^{\text{Q}}$ (*n* = 1–11; *Q* = 0, ±1) clusters of silicon-magnesium sensor material, we calculate NCP and NEC for the ground state structures of Si_2_${\text{Mg}}_{\text{n}}^{\text{Q}}$ (*n* = 1–11; *Q* = 0, ±1), and the results are summarized in the Tables 3–6. We can find that the charges of silicon atoms in Si_2_${\text{Mg}}_{\text{n}}^{\text{Q}}$ (*n* = 1–11; *Q* = 0, ±1) clusters is very significant from the Tables 3–5. Specifically, except for Si_2_${\text{Mg}}_{1}^{+1}$, silicon atoms are negatively charged in the range of −0.34 to −2.06 electrons, and most magnesium atoms are positively charged in the range of 0.02–0.99 electrons. This result is consistent with expectation, because electrons are always transferred from magnesium atoms to silicon atoms in Si_2_${\text{Mg}}_{\text{n}}^{\text{Q}}$ clusters. In short, the NCP of Si atoms indicates that silicon atoms are electron acceptors in Si_2_${\text{Mg}}_{\text{n}}^{\text{Q}}$ clusters. The NEC of silicon atoms can be found in the Table 6, the electronic configuration for silicon atoms (3s^1^3p^3^) shows that 3p orbital get 0.10–2.28 electrons, while 3s orbital loses 0.11–0.47 electrons. Obviously, charge transfer occurs only in the outermost electron orbit, and strong s-p hybridizations are presented in silicon atoms of Si_2_${\text{Mg}}_{\text{n}}^{\text{Q}}$ clusters. Notably, the contributions of 4s and 5d orbitals are almost zero and can be ignored. Moreover, the charges of 3s and 3p orbitals for two silicon atoms in the ground state of Si_2_${\text{Mg}}_{\text{n}}^{\text{Q}}$ clusters are equal except for ${\text{Si}}_{2}{\text{Mg}}_{3-5}^{-}$, ${\text{Si}}_{2}{\text{Mg}}_{7}^{-}$, ${\text{Si}}_{2}{\text{Mg}}_{9-11}^{-}$ ${\text{Si}}_{2}{\text{Mg}}_{4}^{-}$, ${\text{Si}}_{2}{\text{Mg}}_{7-11}^{-}$, Si_2_${\text{Mg}}_{5}^{+}$, and Si_2_${\text{Mg}}_{8-9}^{+}$.

**Table 3:** NCP of the lowest-energy structures for neutral SiMg_*n*_ (*n* = 1–11) clusters of silicon-magnesium sensor material.

**Clusters/Atom**	**Si-1**	**Si-2**	**Mg-1**	**Mg-2**	**Mg-3**	**Mg-4**	**Mg-5**	**Mg-6**	**Mg-7**	**Mg-8**	**Mg-9**	**Mg-10**	**Mg-11**
Si_2_Mg_1_	−0.36	−0.36	0.72										
Si_2_Mg_2_	−0.55	−0.55	0.55	0.55									
Si_2_Mg_3_	−0.88	−0.89	0.45	0.45	0.86								
Si_2_Mg_4_	−0.59	−1.14	0.57	0.29	0.29	0.57							
Si_2_Mg_5_	−0.92	−0.76	0.26	0.27	0.23	0.46	0.45						
Si_2_Mg_6_	−0.89	−0.89	0.23	0.42	0.42	0.00	0.23	0.49					
Si_2_Mg_7_	−1.34	−0.68	0.43	0.22	0.15	0.45	0.52	0.00	0.25				
Si_2_Mg_8_	−1.89	−1.89	0.51	0.59	0.30	0.51	0.51	0.51	0.59	0.25			
Si_2_Mg_9_	−1.37	−1.53	0.61	0.25	0.22	0.42	0.49	0.25	0.22	−0.04	0.49		
Si_2_Mg_10_	−1.69	−1.61	0.49	0.00	0.16	0.34	0.37	0.35	0.37	0.48	0.15	0.59	
Si_2_Mg_11_	−1.70	−1.73	0.37	0.52	0.41	0.30	0.04	0.53	−0.03	0.30	0.22	0.58	0.19

**Table 4:** NCP of the lowest-energy structures for anionic Si_2_${\text{Mg}}_{\text{n}}^{-1}$ (*n* = 1–11) clusters of silicon-magnesium sensor material.

**Clusters/Atom**	**Si-1**	**Si-2**	**Mg-1**	**Mg-2**	**Mg-3**	**Mg-4**	**Mg-5**	**Mg-6**	**Mg-7**	**Mg-8**	**Mg-9**	**Mg-10**	**Mg-11**
${\text{Si}}_{2}{\text{Mg}}_{1}^{-}$	−0.73	−0.73	0.46										
${\text{Si}}_{2}{\text{Mg}}_{2}^{-}$	−0.81	−0.81	0.31	0.31									
${\text{Si}}_{2}{\text{Mg}}_{3}^{-}$	−0.88	−0.88	0.38	0.19	0.19								
${\text{Si}}_{2}{\text{Mg}}_{4}^{-}$	−0.65	−1.07	0.27	0.09	0.09	0.27							
${\text{Si}}_{2}{\text{Mg}}_{5}^{-}$	−0.98	−0.98	0.23	0.07	0.23	0.35	0.07						
${\text{Si}}_{2}{\text{Mg}}_{6}^{-}$	−0.53	−0.53	−0.07	0.08	0.08	−0.07	0.03	0.03					
${\text{Si}}_{2}{\text{Mg}}_{7}^{-}$	−0.79	−1.46	0.22	0.05	0.35	0.19	0.09	−0.02	0.37				
${\text{Si}}_{2}{\text{Mg}}_{8}^{-}$	−1.47	−1.49	0.30	0.27	0.17	0.39	0.19	0.21	0.17	0.26			
${\text{Si}}_{2}{\text{Mg}}_{9}^{-}$	−1.52	−1.67	0.41	0.27	0.04	0.29	0.00	0.33	0.38	0.34	0.12		
${\text{Si}}_{2}{\text{Mg}}_{10}^{-}$	−1.83	−1.74	0.46	0.05	0.02	0.31	0.30	0.31	0.30	0.38	0.02	0.43	
${\text{Si}}_{2}{\text{Mg}}_{11}^{-}$	−1.88	−1.80	0.35	−0.19	0.44	0.45	0.04	0.09	0.04	0.43	0.49	0.30	0.25

**Table 5:** NCP of the lowest-energy structures for cationic Si_2_${\text{Mg}}_{n}^{+1}$ (*n* = 1–11) clusters of silicon-magnesium sensor material.

**Clusters/Atom**	**Si-1**	**Si-2**	**Mg-1**	**Mg-2**	**Mg-3**	**Mg-4**	**Mg-5**	**Mg-6**	**Mg-7**	**Mg-8**	**Mg-9**	**Mg-10**	**Mg-11**
Si_2_${\text{Mg}}_{1}^{+}$	0.02	0.03	0.95										
Si_2_${\text{Mg}}_{2}^{+}$	−0.34	−0.34	0.84	0.84									
Si_2_${\text{Mg}}_{3}^{+}$	−0.38	−0.37	0.55	0.65	0.55								
Si_2_${\text{Mg}}_{4}^{+}$	−1.16	−1.16	0.99	0.99	0.67	0.67							
Si_2_${\text{Mg}}_{5}^{+}$	−1.37	−1.29	0.67	0.66	0.64	1.09	0.61						
Si_2_${\text{Mg}}_{6}^{+}$	−1.15	−1.15	0.44	0.44	0.78	0.44	0.44	0.78					
Si_2_${\text{Mg}}_{7}^{+}$	−1.09	−1.09	0.65	0.42	0.42	0.42	0.42	0.21	0.65				
Si_2_${\text{Mg}}_{8}^{+}$	−1.33	−1.14	0.35	0.49	0.85	−0.12	0.43	0.81	0.30	0.36			
Si_2_${\text{Mg}}_{9}^{+}$	−1.43	−1.49	0.55	0.55	0.35	0.53	0.53	0.19	0.19	0.69	0.35		
Si_2_${\text{Mg}}_{10}^{+}$	−1.51	−1.51	0.49	0.38	0.48	0.40	0.22	0.41	0.48	0.49	0.41	0.26	
Si_2_${\text{Mg}}_{11}^{+}$	−2.06	−2.06	0.12	0.45	0.59	0.66	0.52	0.52	0.59	0.28	0.28	0.45	0.66

**Table 6:** NEC of the lowest-energy structures for neutral and charged Si_2_${\text{Mg}}_{\text{n}}^{\text{Q}}$(*n* = 1–11; *Q* = 0, ±1) clusters of silicon-magnesium sensor material.

**Clusters**	**Neutral**		**Anionic**		**Cationic**	
	**Si-1**	**Si-2**	**Si-1**	**Si-2**	**Si-1**	**Si-2**
Si_2_Mg_1_	3s^1.75^3p^2.60^	3s^1.75^3p^2.59^	3s^1.89^3p^2.83^	3s^1.89^3p^2.83^	3s^1.85^3p^2.11^	3s^1.85^3p^2.10^
Si_2_Mg_2_	3s^1.76^3p^2.77^	3s^1.76^3p^2.77^	3s^1.71^3p^3.07^	3s^1.71^3p^3.07^	3s^1.81^3p^2.51^	3s^1.81^3p^2.51^
Si_2_Mg_3_	3s^1.68^3p^3.19.^	3s^1.67^3p^3.19^	3s^1.65^3p^3.21^	3s^1.65^3p^3.21^	3s^1.73^3p^2.62^	3s^1.73^3p^2.62^
Si_2_Mg_4_	3s^1.69^3p^2.88.^	3s^1.63^3p^3.48^	3s^1.66^3p^2.96^	3s^1.59^3p^3.45^	3s^1.74^3p^3.40^	3s^1.74^3p^3.40^
Si_2_Mg_5_	3s^1.59^3p^3.311^	3s^1.62^3p^3.11^	3s^1.62^3p^3.32^	3s^1.62^3p^3.32^	3s^1.71^3p^3.64^	3s^1.72^3p^3.55^
Si_2_Mg_6_	3s^1.61^3p^3.25^	3s^1.61^3p^3.25^	3s^1.59^3p^3.39^	3s^1.59^3p^3.39^	3s^1.67^3p^3.45^	3s^1.67^3p^3.45^
Si_2_Mg_7_	3s^1.54^3p^3.76^	3s^1.62^3p^3.03^	3s^1.59^3p^3.17^	3s^1.53^3p^3.89^	3s^1.61^3p^3.44^	3s^1.61^3p^3.44^
Si_2_Mg_8_	3s^1.64^3p^4.24^	3s^1.64^3p^4.24^	3s^1.60^3p^3.83^	3s^1.59^3p^3.85^	3s^1.62^3p^3.68^	3s^1.65^3p^3.46^
Si_2_Mg_9_	3s^1.63^3p^3.72^	3s^1.60^3p^3.92^	3s^1.62^3p^3.88^	3s^1.59^3p^4.07^	3s^1.60^3p^3.80^	3s^1.56^3p^3.89^
Si_2_Mg_10_	3s^1.59^3p^4.09^	3s^1.62^3p^3.98^	3s^1.60^3p^4.21^	3s^1.61^3p^4.11^	3s^1.60^3p^3.87^	3s^1.60^3p^3.87^
Si_2_Mg_11_	3s^1.61^3p^4.08^	3s^1.59^3p^4.13^	3s^1.59^3p^4.28^	3s^1.58^3p^4.21^	3s^1.61^3p^4.43^	3s^1.61^3p^4.43^

### Ionization Potential and Electron Affinity of Si_2_${\text{Mg}}_{\text{n}}^{\text{Q}}$(*n* = 1–11; *Q* = 0, ±1) Clusters of Silicon-Magnesium Sensor Material

Adiabatic ionization potential (AIP), vertical ionization potential (VIP), adiabatic electron affinity (AEA), and vertical electron affinity (VEA) are important characteristics of the electronic properties for clusters. On the basis of optimizing the structure, AIP, VIP, AEA, and VEA are calculated and listed in the Table 7 with the following formulas (Deka et al., 2014):

(6) $$AIP=E_{\text{(optimized cation)}}-E_{\left(\text{optimized neutral}\right)}$$

(7) $$VIP=E_{\text{(cation at optimized neutral geometry)}}-E_{\left(\text{optimized neutral}\right)}$$

(8) $$AEA=E_{\left(\text{optimized neutral}\right)}-E_{\text{(optimized anion)}}$$

(9) $$VEA=E_{\left(\text{optimized neutral}\right)}-E_{\text{(anion at optimized neutral geometry)}}$$

**Figure 5:**
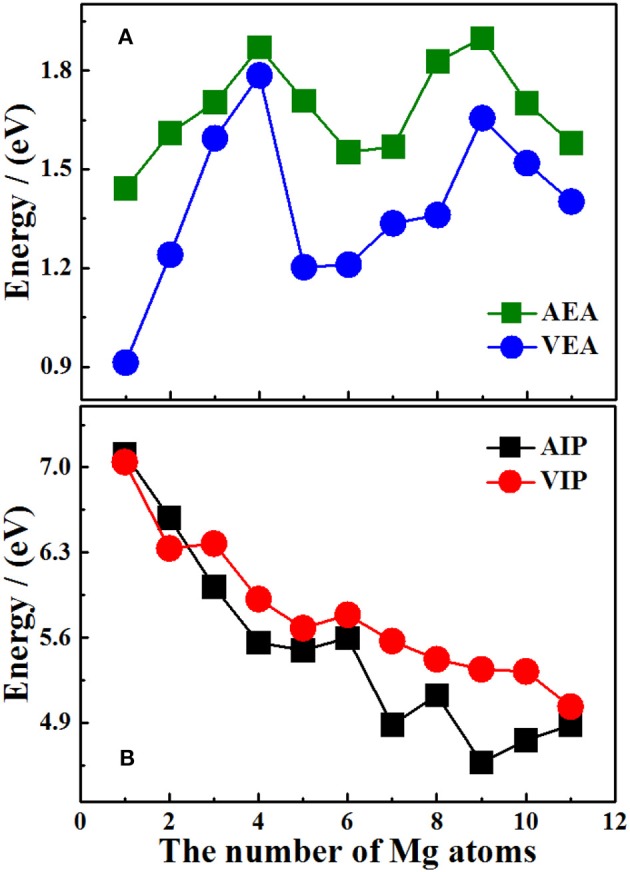
Size dependence of AIP, VIP, AEA, and VEA of ground state Si_2_${\text{Mg}}_{\text{n}}^{\text{Q}}$ (*n* = 1–11; *Q* = 0, ±1) clusters of silicon-magnesium sensor material. **(A)** Size dependent properties of AEA and VEA of the ground state of Si_2_${\text{Mg}}_{\text{n}}^{\text{Q}}$ (Q = 0, ±1; *n* = 1–11) clusters. **(B)** Size dependent properties of AIP and VIP of the ground state of Si_2_${\text{Mg}}_{\text{n}}^{\text{Q}}$(Q = 0, ±1; *n* = 1–11) clusters.

It should be pointed out that the properties of neutral clusters are related to the values of VIP and VEA, while the properties of anionic and cationic clusters are related to AEA and AIP. Figures 5A,B show the size dependence of the AIP, VIP, AEA, and VEA. As Figure 5A showed, the curves of AIP and VIP have the same tendencies as the cluster size increases except *n* = 3, 8, 10. This result means that most cations are similar to the corresponding neutrals. In addition, from the Table 7, we can find that except for *n* = 3, 4, 7, 9, 10, the |AIP-VIP| values are in the range of 0.07–0.29 eV, which implies that the deformation of these structures corresponding to their neutral clusters are not big. The relation between AEA and VEA is showed in the Figure 5B, one can find that they also have the same tendencies and the |AEA-VEA| values are all small except for *n* = 1, 2, 5, 6, and 8, which means that these structures of Si_2_${\text{Mg}}_{\text{n}}^{-1}$ clusters do not differ greatly from the corresponding Si_2_Mg_n_ clusters. In addition, as one knows that |VIP-VEA| can present the chemical hardness and is always used to characterize the stability of clusters (Pearson, 1997). Table 7 also shows the hardness of Si_2_Mg_n_ (*n* = 1–11) clusters, and one can find that the hardness of Si_2_Mg_n_ clusters decreases with the increase of magnesium atoms. It is noteworthy that when *n* = 6, the hardness of the corresponding clusters is obviously larger than that of the adjacent clusters, which indicates that the stability of Si_2_Mg_6_ is higher. This conclusion is consistent with that of the Δ_2_*E* in Figure 4.

**Table 7:** AIP, VIP, AEA, VEA of ground state Si_2_${\text{Mg}}_{\text{n}}^{\text{Q}}$ (*n* = 1–11; *Q* = 0, ±1) clusters of silicon-magnesium sensor material.

***n***	**AIP** **(eV)**	**VIP** **(eV)**	**|AIP-VIP|** **(eV)**	**AEA** **(eV)**	**VEA** **(eV)**	**|AEA-VEA|** **(eV)**	**|VIP-VEA|** **(eV)**
1	7.11	7.04	0.07	1.44	0.91	0.53	6.13
2	6.59	6.33	0.25	1.61	1.24	0.37	5.09
3	6.02	6.37	0.36	1.70	1.59	0.11	4.78
4	5.56	5.92	0.36	1.87	1.78	0.08	4.14
5	5.50	5.68	0.18	1.71	1.20	0.50	4.48
6	5.60	5.79	0.19	1.55	1.21	0.34	4.58
7	4.88	5.57	0.69	1.57	1.34	0.23	4.23
8	5.13	5.42	0.29	1.83	1.36	0.47	4.06
9	4.57	5.34	0.77	1.90	1.66	0.24	3.68
10	4.76	5.32	0.57	1.70	1.52	0.18	3.80
11	4.88	5.03	0.15	1.58	1.40	0.18	3.63

### Infrared and Raman Spectra of Si_2_${\text{Mg}}_{\text{n}}^{\text{Q}}$(*n* = 1–11; *Q* = 0, ±1) Clusters of Silicon-Magnesium Sensor Material

**Figure 6:**
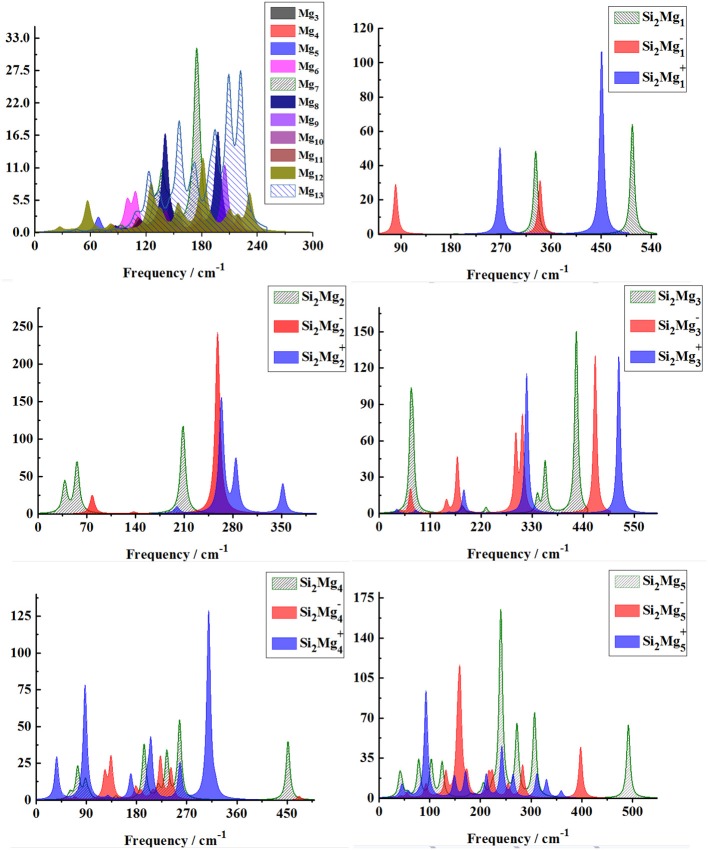
Infrared spectra of the lowest-energy structure of Mg_n+2_ clusters (*n* = 1–11) and Si_2_${\text{Mg}}_{\text{n}}^{\text{Q}}$ (*n* = 1–5; *Q* = 0, ±1) clusters of silicon-magnesium sensor material calculated at B3LYP/6-311G (d) level. Horizontal axes is wave number; vertical axes is IR intensity, km/mol.

**Figure 7:**
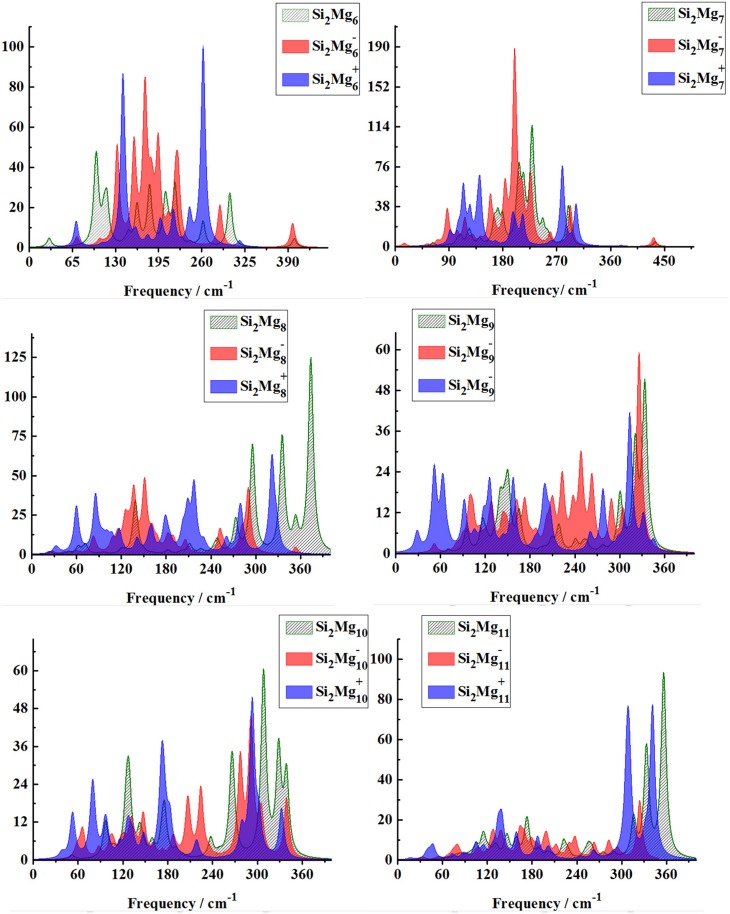
Infrared spectra of the lowest-energy structure of Si2${\text{Mg}}_{\text{n}}^{\text{Q}}$ (*n* = 6–11; *Q* = 0, ±1) clusters of silicon-magnesium sensor material calculated at B3LYP/6-311G (d) level. Horizontal axes is wave number; vertical axes is IR intensity, km/mol.

**Figure 8:**
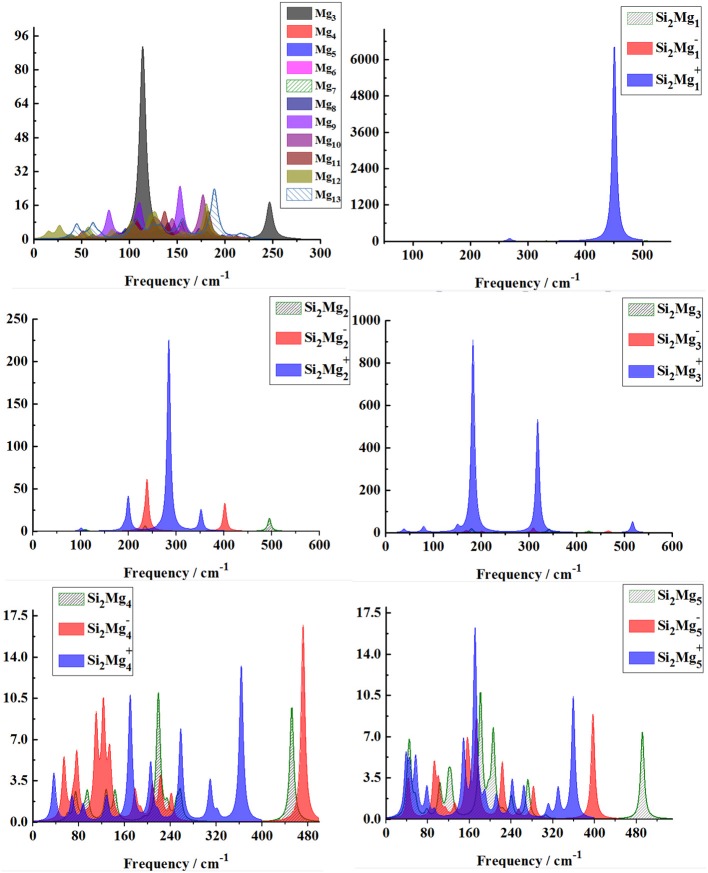
Raman spectra of the lowest-energy structure of Mg_n+2_ clusters (*n* = 1–11) and Si_2_${\text{Mg}}_{\text{n}}^{\text{Q}}$ (*n* = 1–5; *Q* = 0, ±1) clusters of silicon-magnesium sensor material calculated at B3LYP/6-311G (d) level. Horizontal axes is wave number; vertical axes is Raman activity, A^4^/AMU.

**Figure 9:**
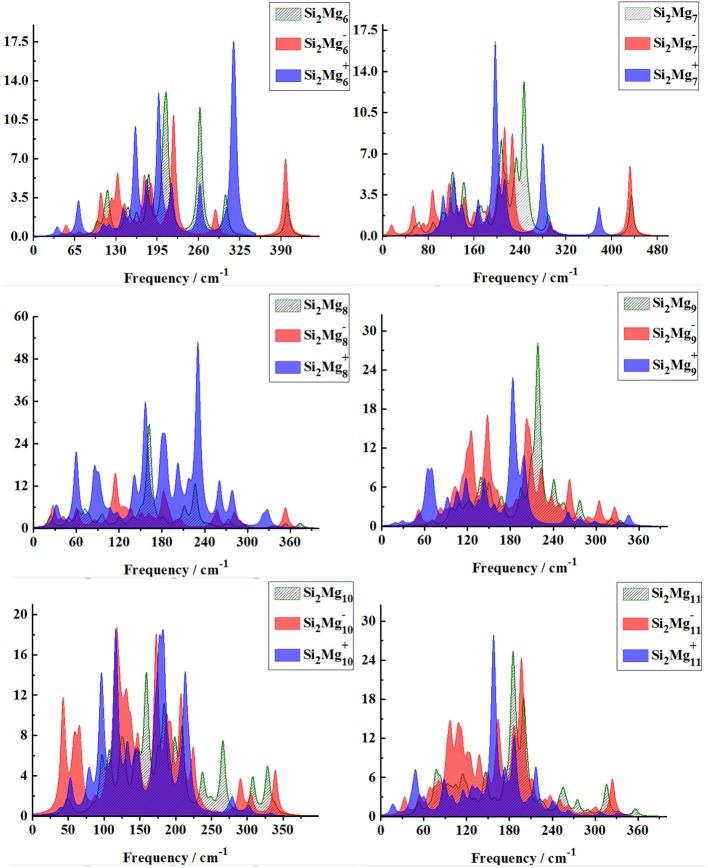
Raman spectra of the lowest-energy structure of Si_2_${\text{Mg}}_{\text{n}}^{\text{Q}}$ (*n* = 6–11; *Q* = 0, ±1) clusters of silicon-magnesium sensor material calculated at B3LYP/6-311G (d) level. Horizontal axes is wave number; vertical axes is Raman activity, A^4^/AMU.

In order to further determine the stability of silicon-magnesium semiconductor sensor material, we calculate the infrared and Raman spectra of ground state of pure Mg_n+2_ and all Si_2_${\text{Mg}}_{\text{n}}^{\text{Q}}$ (*n* = 1–11; *Q* = 0, ±1) clusters at B3LYP/6-311G (d) level, and present them in Figures 6–**9**. Figure 6 presents the infrared spectra of the lowest energy structure of Mg_n+2_ (*n* = 1–11) and Si_2_${\text{Mg}}_{\text{n}}^{\text{Q}}$ (*n* = 1–5; *Q* = 0, ±1) clusters. It is necessary to point out that the vibration spectra (intensity ratio, line width, wave number, and location) are related to the calculation methods and basis groups. For example, the IR spectra of Mg_2−31_ clusters are calculated and showed by two different basis sets under B3PW1 function (Belyaev et al., 2016), but the overall trend of the spectra is similar. By our calculation, the main absorption bands of Mg_n+2_ clusters (*n* = 1–11) are located at 60–230 cm^−1^, which is similar as the results of the existing report (Belyaev et al., 2016). From Figures 6, 7, one can find that the IR strong peaks frequencies are in the range of 40–500 cm^−1^ for neutral Si_2_${\text{Mg}}_{\text{n}}^{0}$ clusters, 80–460 cm^−1^ for anionic Si_2_${\text{Mg}}_{\text{n}}^{-1}$ clusters and 30–540 cm^−1^. In small size (n ≤ 5) clusters, the IR strong vibration spectra of neutral, anionic and cationic Si_2_${\text{Mg}}_{\text{n}}^{\text{Q}}$(*n* = 1–11; *Q* = 0, ±1) clusters are easily distinguished from each other. While, in large size (*n* = 6–11) clusters, the frequency of IR strong vibration spectra of these clusters is relatively close from mid-frequency to the high-frequency region. As we know that the electron-absorbing base moves the infrared absorption peak to the high frequency region, and the electron-supplying base moves the infrared absorption peak to the low frequency region. In addition, the tension property of materials shows that the larger the tension of structures, the higher the infrared absorption frequency. Therefore, we can find that two interesting conclusions from Figures 6, 7. (i) The electron-absorbing base structure of neutral cluster materials is stronger than that of charged clusters, and this trend decreases with the increase of the number of magnesium atoms. (ii) With the increase of magnesium atoms around silicon atoms, the peak infrared absorption frequency shifts from relative high frequency to relative low frequency. This indicates that the tension properties of cluster materials with high Mg atoms are not good. The vibration modes of IR spectra of Si_2_${\text{Mg}}_{\text{n}}^{\text{Q}}$(*n* = 1–11; *Q* = 0, ±1) clusters are very numerous and complex, and as the results discussed above show that magic number clusters of Si_2_${\text{Mg}}_{3}^{0}$, Si_2_${\text{Mg}}_{3}^{-1}$, Si_2_${\text{Mg}}_{3}^{+1}$ are more stable than other clusters. Therefore, here we only focus on these three clusters' vibration modes. As Figure 6 showed, the highest intensity IR frequency of neutral Si_2_${\text{Mg}}_{3}^{0}$ locates at 425.28 cm^−1^, and its vibration mode is assigned as stretching of Si2-Si1 bond. The frequency of the strongest peak of anionic Si_2_${\text{Mg}}_{3}^{-1}$ cluster at 465.87 cm^−1^, and its vibrational mode is as the same as the highest peak of neutral Si_2_${\text{Mg}}_{3}^{0}$. The strong peaks of IR spectra of cationic Si_2_${\text{Mg}}_{3}^{+1}$ cluster at 516.55 cm^−1^ resulted from the stretching of Si2-Si1 bond.

From Figures 8, 9, one can find Raman spectra of Mg_n+2_ and Si_2_${\text{Mg}}_{\text{n}}^{\text{Q}}$ (*n* = 1–11; *Q* = 0, ±1) clusters. Raman spectra activity of Mg_n+2_ (*n* = 1–11) clusters show a fairly low frequency (in the range of 25–180 cm^−1^) nature except for Mg_3_. Raman spectra activity properties of Si_2_${\text{Mg}}_{\text{n}}^{\text{Q}}$ (*n* = 1–11; *Q* = 0, ±1) clusters are rather different from their IR absorption properties. In small size clusters (*n* = 1–3), the Raman activity of cationic Si_2_${\text{Mg}}_{\text{n}}^{\text{Q}}$ clusters is fairly high in Mid-frequency and high-frequency regions. When *n* = 4, 5, the Raman activity of the clusters is widely distributed, and it is easy to distinguish them from each other. However, after *n* > 5, the Raman activity of the clusters begin to shift slowly from the high-frequency region to the mid-frequency region and close to each other. The Raman activity frequency of Si_2_${\text{Mg}}_{\text{n}}^{\text{Q}}$ (*Q* = 0, ±1) clusters are 50–480 cm^−1^ for neutral Si_2_${\text{Mg}}_{\text{n}}^{0}$, 40–480 cm^−1^ for anionic Si_2_${\text{Mg}}_{\text{n}}^{-1}$ and 40–450 cm^−1^, respectively. When studying the vibration information of Raman spectra with specific magic number structure, we can find that the maximum Raman activity of neutral Si_2_${\text{Mg}}_{3}^{0}$ cluster at the frequency of 179.66 cm^−1^ with the stretching of Mg3-Mg4 bond, the frequency of the highest peak of anionic Si_2_${\text{Mg}}_{3}^{-1}$ cluster at 308.76 cm^−1^ is assigned as stretching of Si1-Mg3 and Si2-Mg3 bonds and the highest Raman activity frequency peak of cationic Si_2_${\text{Mg}}_{3}^{+1}$ cluster at 182.25 cm^−1^ vibrated as stretching of Si1-Mg4, Si2-Mg4 bonds.

## Conclusion

The structural, stability, electronic structure and spectral properties of silicon-magnesium semiconductor sensor materials are systematically studied by Si_2_${\text{Mg}}_{\text{n}}^{\text{Q}}$ (*n* = 1–11; *Q* = 0, ±1) clusters in this paper. By using the CALYPSO searching method and B3LYP at 6-311G (d) basis set of DFT, the results can be summarized below:

- The results of Si_2_${\text{Mg}}_{\text{n}}^{\text{Q}}$ (*n* = 1–11; *Q* = 0, ±1) clusters' structure of silicon-magnesium semiconductor sensor material reveal that only a few of the lowest-energy anionic and cationic geometries are similar as their corresponding neutral ones, most of them are deformation. This conclusion is in good agreement with the changes of their AIP, VIP, AEA, and VEA. |VIP-VEA| values reveal that the hardness of Si_2_Mg_n_ clusters decreases with the increase of magnesium atoms.
- For the stability of Si_2_${\text{Mg}}_{\text{n}}^{\text{Q}}$ (*n* = 1–11; *Q* = 0, ±1) clusters of silicon-magnesium semiconductor sensor materials, the average bonding energy of neutral Si_2_${\text{Mg}}_{\text{n}}^{0}$ clusters are always smaller than the anionic and cationic ones show that attachment or detachment of one electron can enhance chemical stabilities of Si_2_${\text{Mg}}_{\text{n}}^{0}$ clusters. Based on the calculations of *E*_b_, *E*_f_, Δ_2_*E*, and *E*_gap_, we find that Si_2_${\text{Mg}}_{3}^{0}$, Si_2_${\text{Mg}}_{3}^{-1}$, Si_2_${\text{Mg}}_{3}^{+1}$, clusters have stronger stabilities than other clusters.
- The cluster electronic structure of silicon-magnesium semiconductor sensor materials is analyzed. The results of NCP and NEC show that the charges in Si_2_${\text{Mg}}_{\text{n}}^{\text{Q}}$ (*n* = 1–11; *Q* = 0, ±1) clusters transfer from Mg atoms to Si atoms, and the sp hybridization is existed in Si atoms in the clusters.
- The infrared (IR) and Raman spectra of Si_2_${\text{Mg}}_{\text{n}}^{\text{Q}}$ (*n* = 1–11; *Q* = 0, ±1) clusters of silicon-magnesium semiconductor sensor materials show different properties. Both IR and Raman spectra can be easily distinguished each other in small size clusters, however, in large clusters, IR spectra converge and concentrate at high frequencies, while Raman spectra converge and concentrate at mid-frequency region.

## Data Availability Statement

All datasets generated for this study are included in the article/supplementary material.

## Author Contributions

All authors listed have made a substantial, direct and intellectual contribution to the work, and approved it for publication.

### Conflict of Interest

The authors declare that the research was conducted in the absence of any commercial or financial relationships that could be construed as a potential conflict of interest.
